# Recombinant Lassa Virus Expressing Green Fluorescent Protein as a Tool for High-Throughput Drug Screens and Neutralizing Antibody Assays

**DOI:** 10.3390/v10110655

**Published:** 2018-11-20

**Authors:** Yíngyún Caì, Masaharu Iwasaki, Brett F. Beitzel, Shuīqìng Yú, Elena N. Postnikova, Beatrice Cubitt, Lisa Evans DeWald, Sheli R. Radoshitzky, Laura Bollinger, Peter B. Jahrling, Gustavo F. Palacios, Juan C. de la Torre, Jens H. Kuhn

**Affiliations:** 1 Integrated Research Facility at Fort Detrick (IRF-Frederick), National Institute of Allergy and Infectious Diseases (NIAID), National Institutes of Health (NIH), B-8200 Research Plaza, Fort Detrick, Frederick, MD 21702, USA; caiy@niaid.nih.gov (Y.C.); Shuiqing.yu@nih.gov (S.Y.); elena.postnikova2@nih.gov (E.N.P.); lmdewald@gmail.com (L.E.D.); bollingerl@niaid.nih.gov (L.B.); jahrlingp@niaid.nih.gov (P.B.J.); 2 Department of Immunology and Microbial Science, The Scripps Research Institute (TSRI), 10550 North Torrey Pines Rd., La Jolla, CA 92037, USA; miwasaki@biken.osaka-u.ac.jp (M.I.); bcubitt@scripps.edu (B.C.); juanct@scripps.edu (J.C.d.l.T.); 3 United States Army Medical Research Institute of Infectious Diseases (USAMRIID), 1425 Porter Street, Fort Detrick, Frederick, MD 21702, USA; brett.f.beitzel.ctr@mail.mil (B.F.B.); sheli.r.radoshitzky.ctr@mail.mil (S.R.R.); gustavo.f.palacios.ctr@mail.mil (G.F.P.)

**Keywords:** *Arenaviridae*, arenavirus, biosafety level 4, BSL-4, drug screening, Lassa fever, Lassa virus, LASV, mammarenavirus, reverse genetics

## Abstract

Lassa virus (LASV), a mammarenavirus, infects an estimated 100,000–300,000 individuals yearly in western Africa and frequently causes lethal disease. Currently, no LASV-specific antivirals or vaccines are commercially available for prevention or treatment of Lassa fever, the disease caused by LASV. The development of medical countermeasure screening platforms is a crucial step to yield licensable products. Using reverse genetics, we generated a recombinant wild-type LASV (rLASV-WT) and a modified version thereof encoding a cleavable green fluorescent protein (GFP) as a reporter for rapid and quantitative detection of infection (rLASV-GFP). Both rLASV-WT and wild-type LASV exhibited similar growth kinetics in cultured cells, whereas growth of rLASV-GFP was slightly impaired. GFP reporter expression by rLASV-GFP remained stable over several serial passages in Vero cells. Using two well-characterized broad-spectrum antivirals known to inhibit LASV infection, favipiravir and ribavirin, we demonstrate that rLASV-GFP is a suitable screening tool for the identification of LASV infection inhibitors. Building on these findings, we established a rLASV-GFP-based high-throughput drug discovery screen and an rLASV-GFP-based antibody neutralization assay. Both platforms, now available as a standard tool at the IRF-Frederick (an international resource), will accelerate anti-LASV medical countermeasure discovery and reduce costs of antiviral screens in maximum containment laboratories.

## 1. Introduction

Lassa virus (LASV) is a highly virulent US National Institute of Allergy and Infectious Diseases (NIAID) Category A Priority Agent [1], US Select Agent [2], and Risk Group 4 Pathogen [3]. LASV causes Lassa fever (LF), a World Health Organization (WHO) Priority Disease due to its epidemic potential and the lack of medical countermeasures (MCMs) [4].

LF was first described in a hospitalized patient and a caretaker in Lassa, Nigeria, in 1969. Both people became severely ill and subsequently died [5]. Today, LF is a relatively common exotic disease in many areas of sub-Saharan Western Africa (e.g., Guinea, Liberia, Nigeria, Sierra Leone) [6,7]. The overall case-fatality rate of LF is approximately 1% of estimated 100,000–300,000 LASV infections occurring annually [8], but the case-fatality rate of hospitalized patients may surpass 50% (and even 90% in infected pregnant women in the third trimester) [9,10,11]. Hence, the burden on the public health sector is significant. At the end of 2017, the largest LF outbreak on record began in Nigeria and later spread to Benin. By 4 November 2018, 553 cases were laboratory-confirmed (case-fatality rate 25.9%) in Nigeria alone [12,13,14,15,16].

The natural host reservoir of LASV is the Natal mastomys (*Mastomys natalensis*) [17]. Human infection typically occurs via direct or indirect exposure to contaminated rodent blood, excretions, or secretions. Person-to-person transmission is not frequent but possible by direct contact with contaminated bodily fluids [8]. Currently, no licensed vaccines are available to prevent LF, and therapeutic options are limited to off-label use of intravenous or oral ribavirin, which is only partially effective and can cause significant side effects [18,19,20].

LASV is an Old World mammarenavirus (*Bunyavirales*: *Arenaviridae*: *Mammarenavirus*) [21] with a bisegmented RNA genome. Each genome segment (S and L) uses an ambisense coding strategy to direct the synthesis of two proteins from two open reading frames (ORFs) separated by a non-coding intergenic region. The S segment encodes the nucleoprotein (NP) and the viral glycoprotein precursor (GPC). GPC is co-translationally processed by signal peptidase to generate a stable signal peptide (SSP) and post-translationally cleaved by the cellular protease SKI-I/S1P to generate GP1 and GP2 subunits. GP1 and GP2 together with SSP form the mature virion surface glycoprotein (GP) complex that is responsible for receptor recognition and cell entry. The L segment encodes the viral RNA-dependent RNA polymerase (L) and the RING finger protein Z, which functions as a matrix protein for the assembly and budding of infectious virions [22].

To date, efforts to discover MCMs against LASV infection have been conducted by a few maximum containment (biosafety level 4) institutes worldwide [23]. Alternatively, other institutions used LASV surrogates that can be handled in biosafety level 2 containment. These surrogates include pseudotyped retroviruses [24,25,26,27,28,29] or vesicular stomatitis Indiana virus particles [30] containing LASV GP to identify and study inhibitors of LASV cell entry, minigenome assays to study inhibitors of LASV replication and transcription [23,31], and Z-based virion-like particles to study inhibitors of LASV cell egress [32,33]. Recently, Welch et al. established a recombinant LASV encoding a ZsGreen fluorescent reporter protein for antiviral screening [23], but the genetic stability of this virus or its reporter was not determined. Here, we report the generation and characterization of a recombinant LASV encoding a different cleavable reporter, green fluorescent protein (rLASV-GFP). We examined the stability of GFP expression by rLASV-GFP during serial passages in two different cell lines and found that cell choice is an important parameter for drug screenings as it can influence GFP stability. In addition, we present for the first time a straightforward antibody neutralization assay using a reporter-encoding LASV for the detection of LASV-neutralizing antibodies. The rLASV-GFP platform is now a generally available tool for maximum containment/biosafety level 4-based high throughput drug screens and neutralization antibody assays at the US Integrated Research Facility at Fort Detrick (IRF-Frederick)—an international resource facilitating high-consequence viral pathogen research [34].

## 2. Materials and Methods

### 2.1. Cell Lines

Human (*Homo sapiens*) adenocarcinoma alveolar basal epithelial A549 (American Type Culture Collection [ATCC], Manassas, VA; #CCL-185) cells, epithelial Hela cells (ATCC, #CCL-2), and hepatocarcinoma Huh-7 cells (a kind gift from Hideki Ebihara, Rocky Mountain Laboratories, Hamilton, MT, USA), and grivet (*Chlorocebus aethiops*) kidney epithelial Vero cells (ATCC, #CCL-81), and Vero E6 cells (ATCC, #CRL-1586) were grown in Gibco Dulbecco’s modified Eagle’s medium (DMEM) (Thermo Fisher Scientific, Waltham, MA, USA) supplemented with 10% heat-inactivated fetal bovine serum (FBS, Sigma-Aldrich, St. Louis, MO, USA). Syrian golden hamster (*Mesocricetus auratus*) kidney BHK-21 fibroblasts (ATCC, #CCL-10) were grown in Gibco DMEM supplemented with 10% FBS and 5% tryptose phosphate broth (TPB, Thermo Fisher Scientific). All cells were incubated at 37°C in a humidified 5% CO2 atmosphere.

### 2.2. Plasmid Construction

Four plasmids (i.e., pCAGGS-LASV-NP, pCAGGS-LASV-L, mPol-I/LASV-Sag [GenBank #MH358389], mPol-I/LASV-Lag [GenBank #MH358388]) used to generate recombinant wild-type LASV strain Josiah (rLASV-WT) have been described [35]. To create a recombinant LASV strain Josiah expressing GFP, we followed a strategy previously used to generate a recombinant lymphocytic choriomeningitis virus expressing GFP [36]. Briefly, NP ORF in plasmid mPol-I/LASV-Sag was replaced with ORF “GFP-2A-NP” (mPol-I/LASV-Sag/GFP-2A-NP) encoding Emerald GFP fused to the N terminus of NP separated by the 2A self-cleaving peptide sequence (ATNFSLLKQAGDVEENPGP) from porcine teschovirus 1 (PTV-1) (GenBank #MH358390).

### 2.3. Rescue of Recombinant Lassa Viruses

All experiments for the rescue or evaluation of recombinant LASV were performed under maximum containment/biosafety level 4 (BSL-4) conditions in the BSL-4 suites of the NIH/NIAID/DCR Integrated Research Facility at Fort Detrick (IRF-Frederick) [34] following approved standard operating procedures [37,38]. BHK-21 cells (7 × 10^5^ cells/well, 6-well plates from Becton Dickinson Labware, Bedford, MA, USA) were co-transfected with 0.8 µg of pCAGGS-LASV-NP, 1.0 µg of pCAGGS-LASV-L, 1.4 µg of mPol-I/LASV-Lag and 0.8 µg of mPol-I/LASV-Sag or mPol-I/LASV-Sag/GFP-2A-NP with 10 µL of Lipofectamine 2000 (Thermo Fisher Scientific). At 5-h post-transfection (PT), media were replaced with 3 mL of DMEM with 2% FBS and 1X Penicillin/Streptomycin (Thermo Fisher Scientific). At 3 days PT, tissue culture supernatants (TCS) were harvested (passage 0, day 3; P0D3), and 3 mL of fresh medium were added to the cells. At 6-days PT, TCS were harvested (P0D6), and 0.5 mL of TCS were used for exposure of Vero cells. TCS were harvested from Vero cultures after 3 days (passage 1, day-3 post-infection; P1D3 PI).

### 2.4. Lassa Virus Propagation

LASV-Josiah, rLASV-WT, and rLASV-GFP virus stocks were prepared in Vero E6 cells using multiplicities of infection (MOIs) of 0.01 in DMEM supplemented with 2% FBS. Vero E6 cells were exposed to LASV Josiah isolate (a kind gift from USAMRIID, Fort Detrick, Frederick, MD, USA), rLASV-WT, or rLASV-GFP at MOIs of 0.01. At 3 days PI, TCS were harvested.

### 2.5. Lassa Virus Titration

LASV titers were quantified by plaque assay. Briefly, confluent monolayers of Vero cells in 6-well plates were infected with serial dilutions of LASV. After 1 h of incubation at 37°C under gentle rocking every 15 min, 2 mL of a 1:1 primary overlay consisting of 1% SeaKem ME Agarose (Lonza, Rockland, ME, USA) and 2X Gibco modified Eagle’s medium (MEM; Thermo Fisher Scientific) containing 10% FBS and 1% Antibiotic-Antimycotic (Sigma-Aldrich) were added to each well. At 3-days PI, 2 mL of a secondary staining overlay consisting of 0.5 % SeaKem ME Agarose and 0.03% neutral red stain (EMD Millipore, Burlington, MA, USA) were added to each well. After incubation at 37°C for 24 h, plaques were counted manually. To improve identification of plaques by imagery, neutral red-stained plates were fixed with 10% neutral-buffered formalin (NBF, Thermo Fisher Scientific) overnight. The agarose overlay was removed, and the plates were stained with 0.2% crystal violet (Ricca Chemical Company, Arlington, TX, USA).

### 2.6. Lassa Virus Growth Kinetics Comparison

A549 and Vero cells were seeded in collagen-coated 24-well plates (Becton Dickinson Labware) at 2 × 10^5^ cells/well or 96-well plates (Becton Dickinson Labware) at 3 × 10^4^ cells/well. After 1 day, media were removed, and cells were washed once with DMEM without FBS. Cells were then exposed to LASV-Josiah, rLASV-WT, or rLASV-GFP at MOIs of 0.01 or 0.1. After 1 h of incubation at 37°C, viral inocula were removed, and cells were washed twice with DMEM without FBS and then supplemented with DMEM containing 2% FBS. At various times PI, TCS were harvested. Viral titers were determined by plaque assay. Cell plates were fixed for 24 h with 10% NBF to inactivate viruses. Fixed cell plates were then transferred from the BSL-4 to the BSL-2 laboratory, stained with human anti-LASV GP monoclonal antibody 37.2G followed by secondary Alexa Fluor 594-conjugated goat anti-human IgG antibody (Life Technologies, Carlsbad, CA, USA) or with mouse anti-LASV NP monoclonal antibody 100LN IgG2b followed by secondary Alexa Fluor 594-conjugated goat anti-mouse IgG antibody (Life Technologies). Hoechst 33342 dye (Thermo Fisher Scientific) was used to stain cell nuclei. Fluorescent signal images were taken with the Operetta High-Content Imaging System and analyzed by Harmony 3.1 software (Perkin Elmer, Waltham, MA, USA).

### 2.7. Assessment of GFP Stability During Serial Passages of rLASV-GFP

To determine the stability of the inserted GFP ORF, rLASV-GFP was passaged 10 times in both Vero and Vero E6 cells. Briefly, Vero or Vero E6 cells were exposed to rLASV-GFP in 75-cm^2^ flasks (defined as passage 0, P0) at an MOI of 0.01. At 72 h PI, TCS were collected (P1), and virus titers were determined by plaque assay. Fresh Vero or Vero-E6 cells were then exposed to P1 TCS at an MOI of 0.01 to generate P2 TCS. This process was serially repeated until P10. P1 to P10 TCS were used for exposure of Vero and Vero E6 cells at an MOI of 0.01. At 72 h PI, plates were fixed with 10% NBF, and plates were analyzed with the Operetta High-Content Imaging System by measuring the percentage of GFP-positive cells in nine random fields of each plate well.

RNA was extracted from LASV (2.5 × 10^5^ pfu) present in TCS from each passage (P0 through P10) by mixing 250 µL of diluted TCS containing 2.5 × 10^5^ pfu with 750 µL of TRIzol LS, followed by RNA extraction using the Purelink RNA Mini Kit (Thermo Fisher Scientific) according to manufacturer’s instructions. The Superscript III One-Step RT-PCR System with Platinum *Taq* High Fidelity DNA Polymerase Kit (Thermo Fisher Scientific) was used to amplify GFP DNA (828 bp) with primers surrounding the GFP-2A cassette (5′-rL-GFP-F: 5′-ATACAACACAACAATCTGGCG-3′; 3′-rL-GFP-R: 5′-GGATTTTATTTCCTTTGAGGCACT-3′). The NP region (nucleotides 819–1,473, 657-bp stretch) was also amplified as a control (primers 5′-LASV-NP-819: 5′-TGGACACAATCTTTGAGGAGGGA-3′; 3′-LASV-NP-1473: 5′-TTTAGGATGGGATGACTTTGAGTC-3′). PCR products were subjected to electrophoresis on 1% agarose gels (Thermo Fisher Scientific).

### 2.8. Cytotoxicity Assays

Cytotoxicity was determined in mock-exposed cells using the Cell Titer-Glo Luminescent Cell Viability Assay kit (Promega, Madison, WI, USA) according to the manufacturer’s instructions. Briefly, cells were seeded in 96-well solid black opaque plates (Thermo Fisher Scientific). Different concentrations of favipiravir (T705, Selleck Chemicals, Houston, TX, USA) or ribavirin (Sigma-Aldrich) were added to the media. At 48 or 72 h, 100 µL of Cell Titer-Glo reagent were added to each well after the plates equilibrated to room temperature. Luminescence was measured by the Infinite® M1000 Tecan plate reader (Tecan, Morrisville, NC, USA).

### 2.9. rLASV-GFP-based Antiviral Drug Screen

A549, Hela, Huh7, and Vero E6 cells were seeded in 96-well plates at densities of 3 × 10^4^ cells per well and grown overnight at 37°C in a 5% CO_2_ atmosphere. Cells were pre-incubated with different concentrations of favipiravir or ribavirin diluted in DMEM without FBS. Pretreated cells were exposed to rLASV-GFP at an MOI of 0.1 in the continued presence of drugs. After incubation at 37°C for 48 h or 72 h, cell plates were fixed with 10% NBF for 24 h to inactivate virus before transfer from the BSL-4 to the BSL-2 laboratory. Hoechst 33342 dye was used to stain cell nuclei. The percentage of GFP-positive cells was measured and analyzed with the Operetta High-Content Imaging System.

### 2.10. rLASV-GFP-based Neutralization Assay

A549 and Vero cells were seeded in collagen-coated 96-well plates at 3 × 10^4^ cells/well. About 5,000 pfu of rLASV-GFP were incubated with different concentrations of human monoclonal neutralizing antibody 37.2D or human IgG control (Thermo Fisher Scientific) for 1 h at 37°C. Then, media were removed, and the virion-antibody mixtures were added on the top of the cell monolayers. After 48 h of incubation at 37°C, cell plates were fixed with 10% NBF for 24 h to inactivate virus and then transferred from the BSL-4 to the BSL-2 laboratory. Hoechst 33342 dye was used to stain nuclei. The percentage of GFP-positive cells was measured and analyzed with the Operetta High-Content Imaging System.

### 2.11. Data Analysis

Non-linear regression analysis and curve fitting parameters (four-parameter variable-slope nonlinear regression model) were performed to calculate the half maximal effective concentration (EC_50_; GraphPad Prism Software, La Jolla CA). Error bars of dose-response curves represent the standard deviation of three replicates. The Student’s *t*-test and analysis of variance (ANOVA) test were used to determine significant differences (*p* <0.05) between groups using GraphPad Prism.

### 2.12. Data Availability

The datasets generated during and/or analyzed during the current study are available from the corresponding author on reasonable request.

## 3. Results

### 3.1. Rescue of Recombinant LASV Expressing GFP (rLASV-GFP)

**Figure 1:**
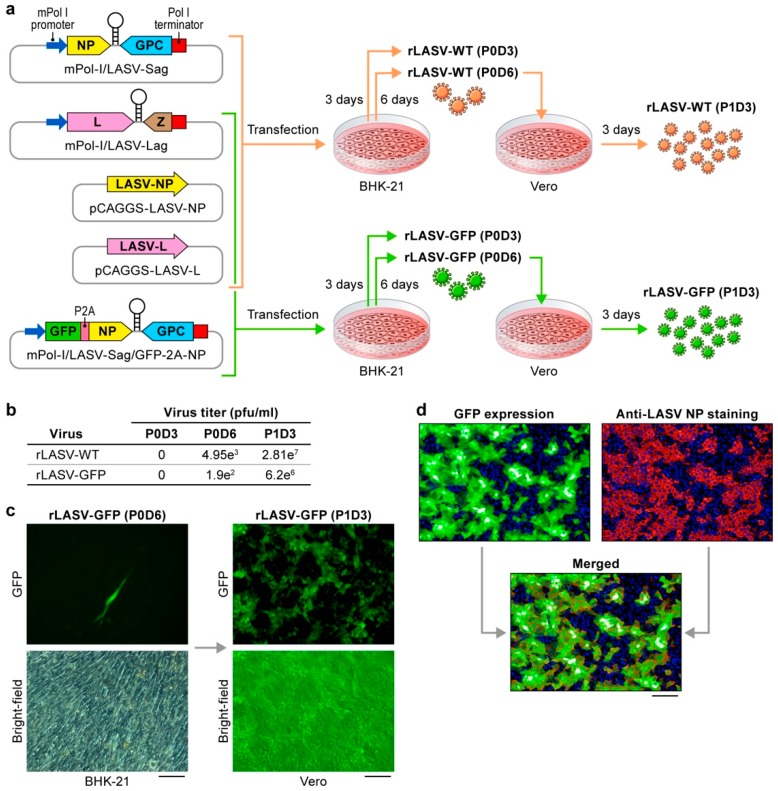
Rescue of recombinant LASV expressing GFP (rLASV-GFP). (**a**) Rescue strategy. Support plasmids pCAGGS-LASV-NP and pCAGGS-LASV-L express LASV nucleoprotein (NP) and viral RNA-dependent RNA polymerase (L), respectively, required for LASV gene transcription and genome replication. Mouse polymerase I promoter (mPol-I)-LASV-Sag and mPol-I-LASV-Lag encode the LASV genomic S and L RNAs segments, respectively. An open reading frame (ORF) encoding GFP was fused to the 3′ end of the ORF encoding NP separated by a sequence encoding the 2A self-cleaving peptide of porcine teschovirus 1 to generate plasmid mPol-I-LASV-Sag/GFP-2A-NP, which was used instead of mPol-I-LASV-Sag to rescue rLASV-GFP. BHK-21 cells were co-transfected with four plasmids as indicated. After 3 days post-transfection (PT), TCS were collected (passage 0, day 3 PT: P0D3), and fresh media were added. After 6 days PT, tissue culture supernatants (TCS) were collected (P0D6) and added to fresh monolayers of Vero cells for another 3 days (P1D3). (**b**) Virus titers in TCS determined by plaque assay. (**c**) Fluorescent micrographs of BHK-21 cells transfected with mPol-I-LASV-Sag/GFP-2A-NP, mPol-I-LASV-Lag, and support plasmids (P0D6) and of culture supernatant-exposed Vero cells (P1D3). Upper Panel: GFP expression. Lower Panel: bright field. Bar, 100 µm. (**d**) Fluorescent micrograph of rLASV-GFP-infected Vero cells (green: GFP expression) immunostained with anti-LASV-NP antibody (red). Hoechst 33342 dye (blue) was used to stain cell nuclei. Bar, 100 µm.

Recombinant viruses expressing reporters such as GFP are valuable for the rapid identification of candidate medical countermeasures. To generate a LASV expressing GFP, we used a previously established reverse genetics system for the rescue of recombinant wild-type LASV (rLASV-WT) [39]. This system consists of four plasmids (Figure 1a). We replaced the LASV S RNA segment-encoding plasmid mPol-I-LASV-Sag with a newly created plasmid mPol-I-LASV-Sag/GFP-2A-NP. This plasmid was modeled after a plasmid used to rescue a recombinant lymphocytic choriomeningitis virus expressing GFP (rLCMV-GFP-2A-NP) [36]. mPol-I-LASV-Sag/GFP-2A-NP encodes GFP fused to the N-terminus of LASV NP separated by the 2A self-cleaving peptide of porcine teschovirus 1 to ensure similar protein expression levels of GFP and NP (Figure 1a).

To rescue both wild type (WT) and GFP-expressing rLASVs, we transfected BHK-21 cells with the indicated set of four plasmids (Figure 1a), harvested TCS 3 days PT (passage 0 TCS, P0D3), and added fresh media to transfected cells. On day 6 PT, we harvested TCS and added them to Vero cells for virus amplification (passage 1, P1). At 3 days PI, we determined titers of viruses in TCS from P0D3, P0D6 and P1 by plaque assay (Figure 1b). Viruses could not be detected at P0D3. At P0D6 viral titers from TCS were low, and rLASV-GFP titers were approximately 10-fold lower than that of rLASV-WT (Figure 1b). Low numbers of GFP-expressing cells were observed following transfection of BHK-21 cells with rLASV-GFP plasmids (Figure 1c). Passaging of these TCS on Vero cells resulted in dramatic amplification of both rLASV-WT and rLASV-GFP, again with rLASV-GFP titers lagging behind those of rLASV-WT (Figure 1b,c). As expected, LASV NP could be detected by immunostaining with anti-LASV-NP antibody in GFP-expressing rLASV-GFP-infected Vero cells (Figure 1d). These data confirm the rescue of a viable rLASV-GFP that was slightly impaired compared to rLASV-WT.

### 3.2. Growth Kinetics of Wild-Type (WT) LASV, rLASV-WT, and rLASV-GFP in Cultured Cells

**Figure 2:**
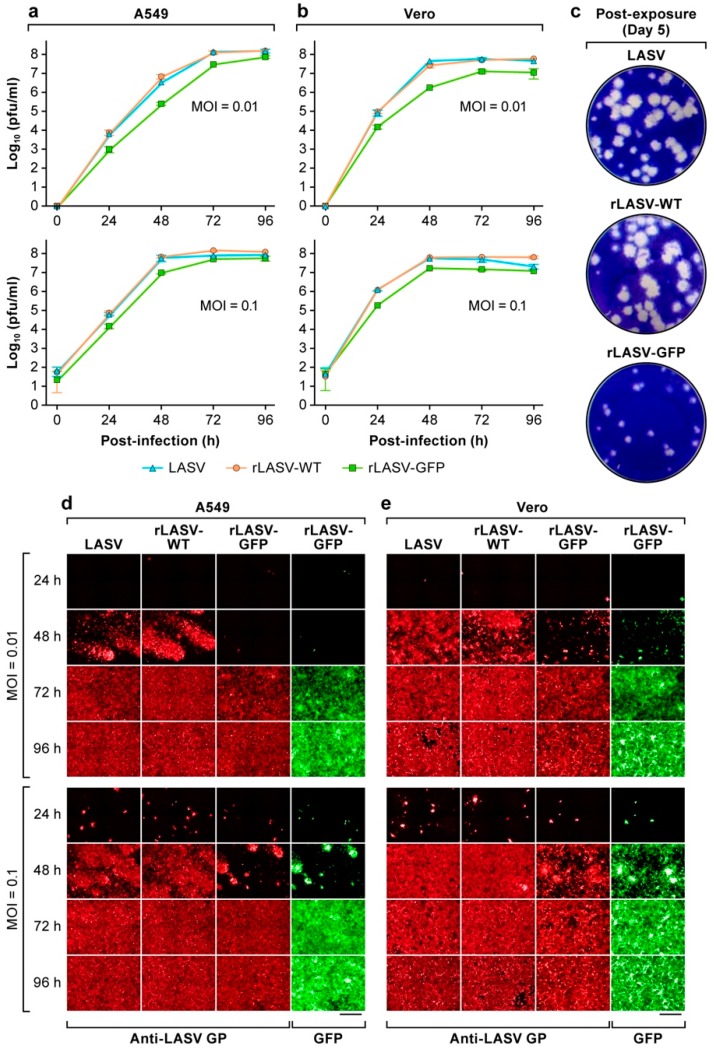
Growth kinetics of wild-type LASV, rLASV-WT, and rLASV-GFP in cultured cells. (**a**) Interferon (IFN)-competent (A549) and (**b**) IFN-deficient (Vero) cells were exposed to all with each virus at MOIs of 0.01 and 0.1. At the indicated time points post-infection (PI), TCS were collected, and virus titers were determined by plaque assay. Graphs represent the means ± standard deviations of triplicate samples. (**c**) Plaque morphologies of each virus on Vero cell monolayers. (**d**,**e**) At the indicated time points PI, A549 and Vero cells were fixed. LASV glycoprotein (GP) was detected (red) by immunofluorescence assay (IFA) using the 37.2G human monoclonal antibody to LASV GP [40] and compared to GFP expression (green). Fluorescence was assessed by high-content imaging. Bar, 200 µm.

We next examined the growth kinetics of LASV, rLASV-WT, and rLASV-GFP in both interferon (IFN)-competent (A549) and IFN-deficient (Vero) cells using MOIs of 0.01 and 0.1. Growth kinetics of LASV and rLASV-WT were nearly identical in both cell lines at both MOIs, reaching peak titers of 1 × 10^8^ plaque-forming units (pfu)/mL. rLASV-GFP also replicated efficiently in both cell lines, although the viral peak titers were about a half log lower than those of LASV or rLASV-WT (Figure 2a,b). Concordantly, the size of the plaques produced by rLASV-GFP was smaller than those caused by LASV and rLASV-WT (Figure 2c). Analysis of the temporal expression of LASV GP as determined by immunofluorescence assay (IFA) confirmed the slower growth kinetics of rLASV-GFP (Figure 2d,e). GFP expression levels correlated with LASV GP expression levels, indicating that the GFP signal may be used to monitor LASV multiplication. These findings demonstrate that the growth of rLASV-GFP is slightly impaired compared to that observed with LASV and rLASV-WT.

### 3.3. Stability of GFP during Serial Passages of rLASV-GFP

**Figure 3:**
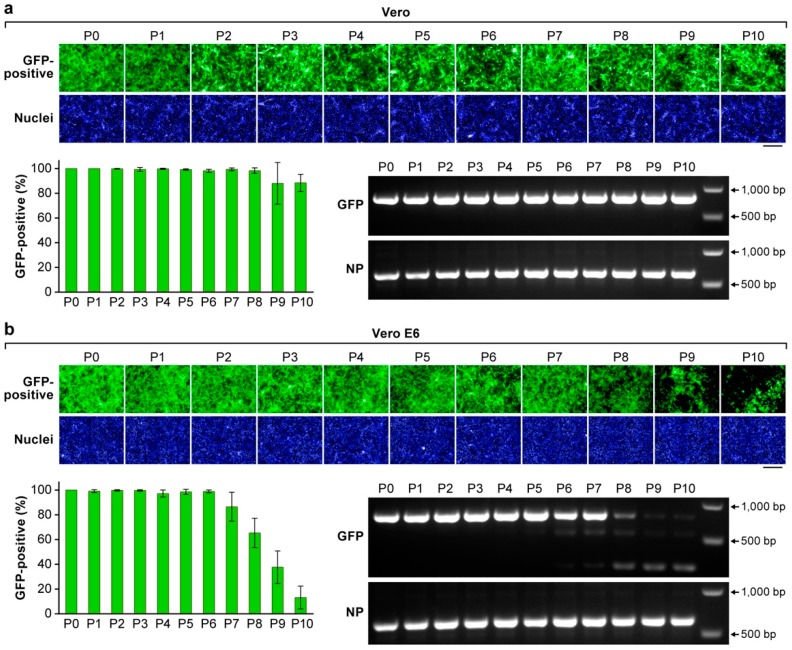
Stability of the GFP reporter gene expressed by rLASV-GFP. Cells were exposed to rLASV-GFP (MOI = 0.01). At 72 h PI, TCS were collected (passage 1, P1), and virus titers were determined by plaque assay. Fresh Vero (**a**) or Vero-E6 cells (**b**) were exposed to with TCS from P1 (MOI = 0.01). This process was serially repeated throughout P10. GFP expression (green) was measured by high-content imaging. Nuclei were stained with DAPI (blue). Viral RNA was extracted from TCS of P1 to P10 to amplify DNA fragments containing an GFP-P2A and a part of the NP ORF using RT-PCR. Images are representative field images of individual wells. Bar, 200 µm.

The reporter stability of rLASV-GFP was investigated by serial passaging of the virus in Vero and Vero E6 cells. Cells were exposed to rLASV-GFP at an MOI of 0.01. At 72 h PI, TCS were collected, and virus titers were determined by plaque assay. Fresh Vero and Vero E6 cell monolayers were infected (MOI = 0.01) with TCS from P1. This process was serially repeated throughout passages P2 to P10. We examined GFP expression for each passage by high-content imaging. In Vero cells, the percentage of GFP-positive cells was consistent from P1 through P10 (Figure 3a). However, in Vero E6 cells, the percentage of GFP-positive cells began to decrease at P7 (90% compared to P0) and was ≈10% at P10 (Figure 3b). To further investigate the reason for the observed loss of GFP expression, we extracted viral RNA from infected cells at P1 to P10 and used RT-PCR to amplify the GFP-2A and NP ORFs. Results from RT-PCR revealed the presence of a deletion within the GFP-2A ORF starting from P6 TCS from Vero E6, but not Vero cells, whereas the NP ORF remained intact (Figure 3a,b). Whether additional specific mutations throughout the rLASV-GFP genome are selected during serial passages in cultured cells remains to be determined.

### 3.4. Antiviral Drug Evaluation Based on rLASV-GFP

**Figure 4:**
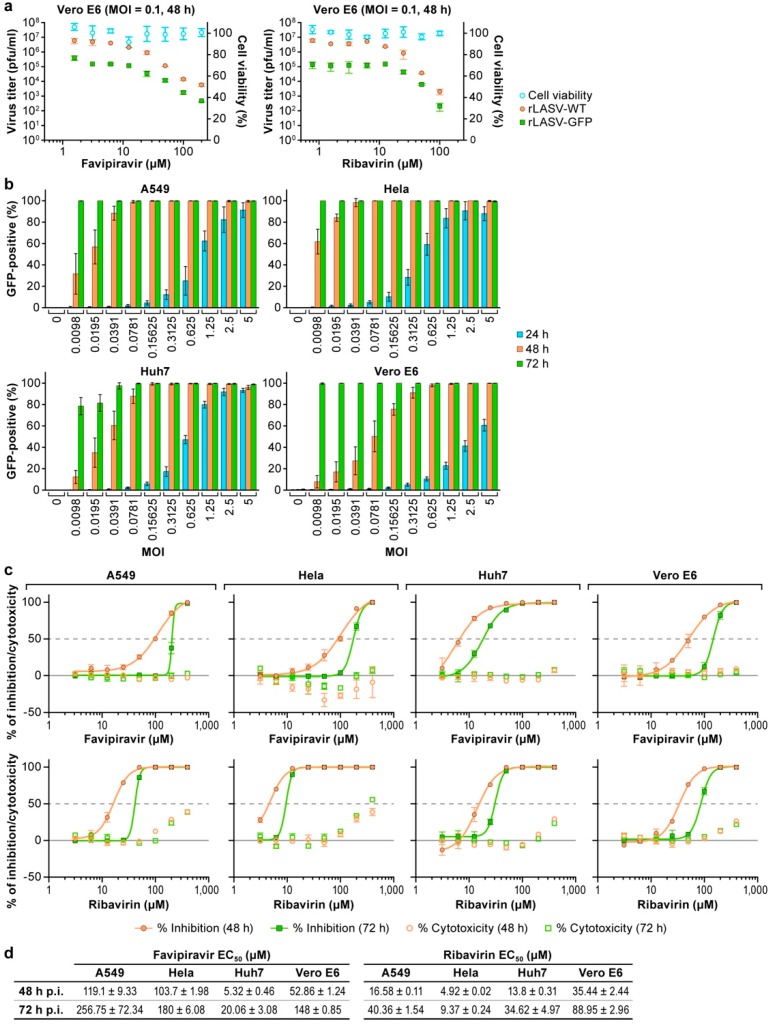
Antiviral drug evaluation based on recombinant LASV expressing GFP (rLASV-GFP). (**a**) Vero E6 cells were pretreated with drugs at the indicated concentrations and then exposed to rLASV-WT or rLASV-GFP (MOI = 0.1) in the presence of the drugs. Viral titers in TCS at 48 h PI were determined by plaque assay. Values represent the means ± standard deviations of triplicate samples. (**b**) Infectivity of rLASV-GFP in A549, HeLa, Huh7, and Vero E6 cells at the indicated MOIs at 24, 48, and 72h PI as determined by GFP-expression using high-content imaging. (**c**) Effect of favipiravir and ribavirin on rLASV-GFP multiplication at 48 (orange filled circles) and 72 h PI (green filled squares). Cells were exposed to rLASV-GFP (MOI = 0.1) and treated with various concentrations of favipiravir or ribavirin. The percentage of GFP-positive cells was determined at 48 h or 72 h PI. (**d**) Half-maximal effective concentrations (EC_50_) of favipiravir and ribavirin to inhibit rLASV-GFP infection in four cell types at 48 and 72 h PI.

To examine whether GFP expression could be used as a surrogate readout for LASV multiplication to rapidly assess the activity of antiviral drug candidates, we examined the sensitivity of rLASV-GFP to favipiravir and ribavirin using a virus yield-based dose response assay. These two broad-spectrum antiviral drugs are known to inhibit LASV replication in vitro and in vivo [20,41,42,43] and, hence, serve as well-vetted positive controls. Vero E6 cells were treated with increasing concentrations of favipiravir or ribavirin 1 h prior infection with rLASV-WT or rLASV-GFP (MOI = 0.1), and at 48 h PI viral titers in TCS were determined by plaque assay. As expected, favipiravir and ribavirin both inhibited rLASV-GFP and rLASV-WT yields in an inverse dose-dependent manner (Figure 4a). The 90% effective concentration (EC_90_) of favipiravir was 30.89 µM for rLASV-WT and 14.10 µM for rLASV-GFP. The EC_90_ of ribavirin was 29.81 µM for rLASV-WT and 6.12 µM for rLASV-GFP.

To confirm the feasibility of using rLASV-GFP to develop a high-throughput drug screening assay, we evaluated the infectivity of rLASV-GFP in four different cell lines (A549, HeLa, Huh7, Vero E6) used for LASV drug evaluations at the IRF-Frederick. Cells were exposed to rLASV-GFP at 10 different MOIs (range: 0.01 to 5). After 24, 48, or 72 h PI, infectivity was measured as percentage of GFP expression using the Operetta High-Content Imaging System. At 24 h PI, the number of GFP-positive cells was overall lower in Vero E6 cells compared to A549, HeLa, or Huh7 cells (Figure 4b). At 72 h PI, the percentage of rLASV-GFP-positive cells in all four cell lines was at least 80% even at the lowest MOI (0.01).

We next used rLASV-GFP to evaluate the antiviral efficacy of favipiravir and ribavirin in all four cell types. Cells were treated with increasing concentrations of favipiravir or ribavirin 1 h prior to exposure to rLASV-GFP (MOI = 0.1). EC_50_ values were consistently higher when determined at 72 h PI compared to that observed at 48 h PI (Figure 4c,d). In this assay, favipiravir was most effective in Huh7 cells (EC_50 (Huh7)_ < EC_50 (Vero E6)_ [*p* < 0.0001] < EC_50 (HeLa)_ [*p* < 0.0001] ≤ EC_50 (A549)_)_,_ whereas ribavirin was most effective in HeLa cells (EC_50 (Hela)_ < EC_50 (Huh7)_ [*p* < 0.0001] ≤ EC_50 (A549)_ < EC_50 (Vero E6)_ [*p* < 0.0001]) (Figure 4c,d)_._ These data indicate that rLASV-GFP can be used to facilitate high-throughput drug screens. However, our findings also indicate that candidate antiviral drugs ought to be evaluated in multiple cell types.

### 3.5. Neutralization Assay Based on rLASV-GFP

**Figure 5:**
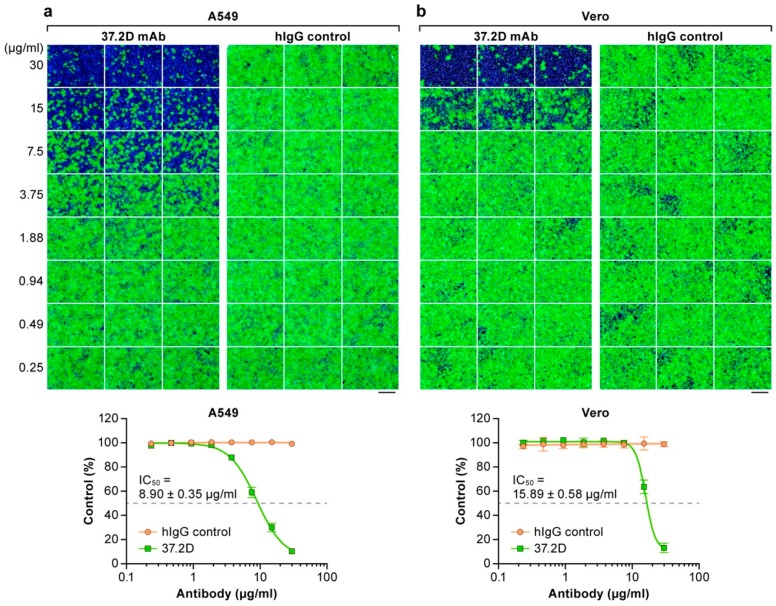
Neutralization assay based on recombinant GFP-expressing LASV (rLASV-GFP). rLASV-GFP was pre-incubated for 1 h with the indicated concentrations of a LASV-neutralizing human monoclonal antibody (37.2D) or human IgG isotype control (hIgG) and then added to A549 (**a**) or Vero (**b**) cells. GFP expression was assessed by high-content imaging. (Top) Representative images. (Bottom) The percentage of rLASV-GFP-infected cells in the presence of 37.2D antibody (green) and hIgG control (orange) was determined by high-content imaging. Error bars indicate the standard deviation of triplicate samples. The IC_50_ for A549 cells was significantly lower than that for Vero cells (Student’s *t*-test, *p* <0.0001). Bar, 100 µm.

To evaluate whether GFP expression by rLASV-GFP could be used to evaluate antibody neutralizing activity, we measured the effect on rLASV-GFP infectivity of the previously identified human monoclonal LASV-neutralizing antibody 37.2D [40,44]. rLASV-GFP was pre-incubated with increasing concentrations of 37.2D antibody or control human IgG at 37°C for 1 h. A549 and Vero cells were exposed to the treated virus. The percentage of infected cells was then measured through GFP expression using high-content imaging. The half-maximal inhibitory concentration (IC_50_) of 37.2D was 8.9 µg/mL in A549 cells and 15.9 µg/ml in Vero cells (Figure 5), i.e., within the range of the previously reported IC_50_ of the 37.2D antibody for LASV (4.36 µg/mL as determined by plaque-reduction neutralization test on Vero 76 cells) [40]. These results demonstrate the feasibility of using rLASV-GFP to assess the neutralizing activity of LASV-specific antibodies.

## 4. Discussion

The recent LF outbreak of unprecedented magnitude in Nigeria and Benin [12,13,14], and the lack of licensed MCMs to counter LASV infection [44] underscore the high priority of establishing methods for the rapid identification of candidate LASV therapeutics. Recombinant LASV expressing reporter proteins such as GFP are powerful tools for the implementation of screens for rapid identification of candidate MCMs. Recently, Welch et al. established a recombinant LASV encoding a ZsGreen fluorescent reporter protein for antiviral screening [23], but the stability of this virus or its reporter was not characterized nor was that virus used for the establishment of a neutralizing antibody assay. ZsGreen and GFP have different fluorescence profiles in animal tissues and cells and, therefore, may have different applications depending on research question [45]. Because GFP is a widely used reporter, we created and characterized a recombinant LASV expressing GFP as an alternative tool to rLASV-ZsGreen for high-throughput drug screens and neutralizing antibody assays. The use of GFP as a readout of virus multiplication can be performed with a fluorescence microscope or Tecan reader. This readout streamlines antiviral drug screens or neutralization assays compared to a virus yield-based dose response assay [46] or a standard plaque-reduction neutralization test (PRNT) [40]. Several surrogate screening systems, including pseudotyped virions carrying LASV GP or LASV minigenome systems [23,24,25,26,27,28,29,30], can only be used to test one specific step of the LASV lifecycle. In contrast, infection with rLASV-GFP or the previously described rLASV-ZsGreen [23] can be used to screen libraries to identify antiviral drug candidates that target any of the steps of the LASV lifecycle (i.e., cell entry, virus RNA replication and gene transcription, virion assembly, cell egress). Indeed, the rLASV-GFP system described here has already been used successfully in two studies supported by the authors to evaluate the effect of small molecule compounds on LASV replication [47,48]. Moreover, rLASV-GFP could also provide a valuable tool for genetic screens including siRNA or CRISPR library screening to identify host cell factors that play a vital role in the LASV lifecycle.

In this study, we evaluated for the first time the stability of rLASV-GFP during serial passages in cultured cells. The GFP ORF proved to be more stable during passages in Vero than in Vero E6 cells. We cannot at this point explain this difference, as Vero E6 cells were derived from Vero cells. We conducted only one serial passage of rLASV-GFP in each cell line, and, hence, the reporter instability of rLASV-GFP in Vero E6 cells plausibly could be a random event. A comprehensive assessment of the stability of the GFP expression during serial passages of rLASV-GFP in VeroE6 cells would require conducting additional independent serial passages. Likewise, whether specific mutations throughout the rLASV-GFP genome are selected during serial passages in cultured cells remains to be determined.

Virus input, duration of infection (experimental endpoint), and cell type affect the measurement of antiviral drug potency. Accordingly, we observed that favipiravir was more effective in Huh7 cells (EC_50_ of ≈5.32 μM at 48 h PI and ≈20.06 μM at 72 h PI) than in Vero E6, HeLa, or A549 cells, whereas ribavirin exerted its strongest effect against rLASV-GFP (MOI = 0.1) infection in HeLa cells (EC_50_ of ≈5.92 μM at 48 h PI and ≈9.37 μM at 72 h PI). In contrast, ribavirin had an EC_50_ of ≈2.47 μM in Huh7 cells at 72 h PI with rLASV-ZsGreen (MOI = 0.1) [23]. In experiments using WT-LASV (strain AV; MOI = 0.01), the EC_50_ of ribavirin was 65.5 μM in Vero cells at 48 h PI [49]; whereas the EC_50_ of favipiravir and ribavirin in Vero E6 cells infected with WT-LASV (strain Ba366; MOI = 0.01) at 72 h PI was 16.6–52.3 μM and 20.9–32.2 μM, respectively [41]. Together, these results underscore the critical importance of re-evaluating the performance of a rLASV expressing a reporter gene in each new cell type. These results are also a reminder that rLASV expressing distinct reporters behave differently and a careful a priori determination of which cell type ought to be used for which kind of inhibitor is needed.
